# Structural basis of the XPB–Bax1 complex as a dynamic helicase–nuclease machinery for DNA repair

**DOI:** 10.1093/nar/gkaa324

**Published:** 2020-05-06

**Authors:** Kevin DuPrez, Feng He, Zhenhang Chen, Eduardo Hilario, Li Fan

**Affiliations:** Department of Biochemistry, University of California, Riverside, CA 92521, USA

## Abstract

Nucleotide excision repair (NER) is a major DNA repair pathway for a variety of DNA lesions. XPB plays a key role in DNA opening at damage sites and coordinating damage incision by nucleases. XPB is conserved from archaea to human. In archaea, XPB is associated with a nuclease Bax1. Here we report crystal structures of XPB in complex with Bax1 from *Archaeoglobus fulgidus* (Af) and *Sulfolobus tokodaii (St)*. These structures reveal for the first time four domains in Bax1, which interacts with XPB mainly through its N-terminal domain. A Cas2-like domain likely helps to position Bax1 at the forked DNA allowing the nuclease domain to incise one arm of the fork. Bax1 exists in monomer or homodimer but forms a heterodimer exclusively with XPB. StBax1 keeps StXPB in a closed conformation and stimulates ATP hydrolysis by XPB while AfBax1 maintains AfXPB in the open conformation and reduces its ATPase activity. Bax1 contains two distinguished nuclease active sites to presumably incise DNA damage. Our results demonstrate that protein-protein interactions regulate the activities of XPB ATPase and Bax1 nuclease. These structures provide a platform to understand the XPB-nuclease interactions important for the coordination of DNA unwinding and damage incision in eukaryotic NER.

## INTRODUCTION

Xeroderma pigmentosum type B (XPB) gene encodes a superfamily 2 (SF2) DNA helicase conserved from archaea to human (1–8). In eukaryotes, XPB is the largest subunit of the general transcription factor TFIIH complex required for both transcription and nucleotide excision repair (NER) (9–11). Due to its biological importance, inherited *xpb* mutations are associated with sensitivity to UV light including sunlight and high risk of skin cancer or developmental disorders (12,13).

NER removes a variety of DNA helix-distortion lesions including cyclobutane pyrimidine dimer (CPD), [6–4] photoproduct (6-4PP), cis-platinum adduct, and bulky chemical adducts caused by carcinogen exposure through a ‘cut and patch’ mechanism (14). NER can be broken down into 4 steps: (1) damage recognition, (2) DNA unwinding around the lesion and damage verification, (3) dual incisions to remove a damage-containing DNA fragment and (4) DNA re-synthesis to fill in the resultant gap. Two distinct NER subpathways have evolved: the transcription coupled repair (TCR) and global genome repair (GGR). These two subpathways differ only in the damage recognition step: TCR is activated upon the stalling of an actively transcribing RNA polymerase II by a lesion in the transcribed DNA strand; whereas GGR utilizes the damage recognition factors XPC-HR23B and UV-DDB to scan the genome for variations in DNA structure and chemistry. Following damage recognition, the two subpathways converge by the recruitment of other NER factors to the damage site, such as TFIIH, XPA and replication protein A (RPA), which together lead to localized unwinding of the DNA around the lesion by the action of the TFIIH helicase subunits XPB and XPD. During DNA unwinding, the engagement of XPD with the damage plays a role in damage verification to facilitate the assembly of a pre-incision complex including two nucleases XPG and the XPF–ERCC1 complex. The DNA is then cleaved at the 5′ and 3′ sides of the lesion by the XPF–ERCC1 complex and XPG, respectively (15–17). The resulting gap in the damaged DNA strand is filled and ligated by coordinated reactions of DNA polymerases (δ, ϵ or κ), replication factor C (RFC), proliferating cellular nuclear antigen (PCNA) and XRCC1–DNA ligase III/α complex or a flap endonuclease 1 (FEN1)–DNA ligase I complex (18–21).

It is believed that XPB is essential for the initial DNA opening at the damage site although XPD is the more robust helicase but requires a ssDNA overhang to start dsDNA unwinding (22–26) through the ‘inch worm’ mechanism (27,28) as a conventional SF2 helicase. XPB has been proposed to function as a ‘molecular wrench’ (29) or dsDNA translocase (30,31) during transcription. Structural analysis on crystal structures of archaeal XPB homologs AfXPB (PDB entry: 2FWR) (1) and StXPB (PDB entry: 5TNU) (32) suggested that domain rotation in XPB may induce a spiral movement on dsDNA (25). This spiral DNA movement causes a supertwist at the promoter bound by other transcription factors leading to promoter melting for transcription initiation. This hypothesis is supported by recent results from DNA unwinding analyses on dsDNA fixed on electrochemical analytical chips (32). Similarly, XPB could create a supertwist at the lesion site bound by the damage recognition complex (33) to initiate DNA opening at the damage site allowing XPD to extend the bubble and verify DNA damage in nucleotide excision repair. The recent cryo-EM structure of XPA bound to TFIIH with a forked DNA substrate clearly showed that human XPB acts as a translocase by binding to the dsDNA region ahead of the fork during DNA repair (34).

Most archaea have homologs of human NER proteins such as XPB, XPD and XPF. Structural and biochemical studies of these archaeal proteins have provided pivotal advances in our understanding of key NER steps (35). Structural studies of archaeal XPD helicases revealed a key FeS domain for DNA binding and a likely role in damage verification (23–25). The crystal structure of AfXPB (1) uncovered a unique RED motif and a thumb (ThM) motif, which were reported later to be essential for the recruitment of human XPB (TFIIH) to the damage site *in vivo* (36). In addition, structural studies on archaeal XPB revealed several domain orientations in XPB (32), suggesting domain rotation induced by ATP binding and hydrolysis could allow XPB to function as a molecular wrench or DNA translocase (25). An XPG-like nuclease, named Bax1, has been reported to form a heterodimer with XPB in many archaea (2,37–40). The *Bax1* gene is in close proximity to the *XPB* gene in euryarchaea (such as *Thermoplasma acidophilum* and *Archaeoglobus fulgidus*) and to the *XPBII* gene in crenarchaea (for example, *Sulfurisphaera (formerly Solfalobus) solfataricus and tokodaii*), which contain two XPB homologs named *XPBI* and *XPBII*. Only the *XPBII* gene product interacts with Bax1. For simplicity and unity, we name crenarchaeal XPBII as XPB in this report. Bax1 has been shown to stimulate the ATPase activity of XPB while XPB plays a role in regulating Bax1 activity by increasing the DNA affinity of Bax1 and influences DNA incision by Bax1. Furthermore, XPB and Bax1 nuclease have been reported to work together to unwind and cleave DNA substrates resembling NER intermediate DNA structures (39). Here, we further characterized the interactions of XPB with Bax1 and the impact on both XPB ATPase and Bax1 nuclease. In addition, we determined the crystal structures of the AfXPB–Bax1 complex and the StXPB–Bax1 complex. These structures provide a platform to understand how XPB and Bax1 function together as a dynamic machinery for DNA unwinding and incision, and have implications for how XPB interacts with nucleases XPG and XPF in eukaryotic NER.

## MATERIALS AND METHODS

### Purification, crystallization, and structural determination of the AfXPB–Bax1 complex

Both AfXPB (Protein Accession number: AAB90879) and AfBax1 (Protein Accession number: WP_010877864) were cloned by PCR using *Archaeoglobus fulgidus* genomic DNA (ATCC) as the template into pET-28b and pET-15b, respectively. His_6_-AfXPB and AfBax1 were expressed under the same conditions: *E. coli* Rosetta (DE3) pLysS cells transformed with pET-28b/AfXPB or pET-15b/AfBax1 were cultured at 28°C with induction by 0.4 mM IPTG, followed by incubation at 28°C for 16–18 h. Cells expressing His_6_-AfXPB were lysed in buffer A (50 mM Tris–Cl pH 7.5, 500 mM NaCl, 5% (v/v) glycerol, 0.01% (w/v) sodium azide) by sonication, followed by heat-denaturation of native *E. coli* proteins at 60–65°C for 10 min. Soluble protein was isolated by centrifugation at 10 000 *g* for 15 min, supplemented with 500 mM imidazole to 30 mM, then loaded onto a 5 ml HisTrap column (GE) equilibrated in buffer A using an ÄKTA prime Plus FPLC (GE). The column was washed with buffer A supplemented with 30 mM imidazole, followed by buffer A2 (50 mM Tris–Cl pH 7.5, 150 mM NaCl, 5% (v/v) glycerol, 0.01% (w/v) sodium azide) supplemented with 30 mM imidazole. Protein was eluted with buffer B (50 mM Tris–Cl pH 7.5, 150 mM NaCl, 5% (v/v) glycerol, 500 mM imidazole, 0.01% (w/v) sodium azide). Peak fractions were combined and diluted 10-fold with buffer A2, then loaded onto 2 × 5 ml HiTrap SP cation-exchange columns (GE) equilibrated in buffer A2. The columns were washed with buffer A2, then the HisTrap column was attached below the SP columns, allowing for eluted protein in buffer B2 (50 mM Tris–Cl pH 7.5, 1.0 M NaCl, 5% (v/v) glycerol, 0.01% (w/v) sodium azide) to bind the HisTrap column. A lysate of non-tagged AfBax1 prepared in the same way as for His_6_-AfXPB was loaded on the AfXPB-bound HisTrap column, with wash and elution steps as for AfXPB alone. Peak fractions were loaded directly onto a HiPrep 16/60 Sephacryl S-200 (GE) gel filtration column equilibrated in buffer D (10 mM Tris–Cl pH 7.5, 500 mM NaCl, 5% (v/v) glycerol, 0.01% (w/v) sodium azide). The peak fractions corresponding to the AfXPB–Bax1 complex were combined and concentrated to 8–10 mg/ml. His_6_-AfXPB was purified alone by essentially the same method above, only the cation-exchange column elution was directly loaded to the gel filtration column, with concentration of peak fractions to ∼10 mg/ml. His_6_-AfBax1 was expressed from pET-28b transformed in *E. coli* Rosetta cells and purified in the same way as for His_6_-AfXPB. Seleno-methionine (Se-Met) derivative AfBax1 was expressed as for the native protein using M9 minimal media prepared via published methods (41), with induction, growth, and purification of the complex with AfXPB carried out as for the native protein. AfXPB N-terminal degradation product and His_6_-AfXPB C-terminal half were both prepared as described (1).

Crystals of the AfXPB–Bax1 complex were obtained by vapor-diffusion from drops composed of a 1:1 mixture of protein with reservoir solution containing 100 mM sodium acetate pH 4.6 and 1800 mM sodium acetate after incubation at room temperature. Crystals were cryo-protected by transferring the cover slips with the crystal-containing drops over solutions of 2800 mM ammonium sulfate to dehydrate the drops. After 1 week, crystals were flash-cooled in a 100 K nitrogen stream and stored in a liquid nitrogen dewar for shipment to synchrotron facilities. Crystals of the Se-Met derivative protein complex were obtained from the same condition, though were cryo-protected by serial washing in mother liquor supplemented with 5, 10 and 15% (v/v) ethylene glycol, then stored as for the native protein crystals.

X-ray diffraction data for the AfXPB–Bax1 and the Se-Met derivative complex was collected at the Advanced Light Source (beamline 12.3.1, SIBYLS), Berkeley, CA, at 100 K. Data for the native complex was collected with 1.0 Å wavelength radiation, while peak, inflection, and remote datasets were collected for the Se-Met derivative complex based on the observed selenium fluorescence to maximize the anomalous difference signal from the Se atoms. X-ray data processing and protein structure refinement was carried out with the associated programs of the CCP4 (42) and PHENIX suites (43). The native protein crystals diffracted in space group C2 with two copies of each protein heterodimer per asymmetric unit; indexing of the Se-Met derivative datasets showed them to be isomorphous with the native crystals. Initial phases for the XPB component were determined by molecular replacement with the *A. fulgidus* XPB N-terminal and C-terminal halves (PDB entry: 2FZ4 and 2FZL, respectively) as search models. The anomalous signal from selenium sites of the AfBax1 Se-Met derivative were used in combination with secondary structure prediction to build the AfBax1 structure. The Rfree statistic was based on 5% of the total reflections and was monitored throughout the refinement. The asymmetric unit contains two copies of the protein heteroduplex consisting of amino acid residues 1 through 445 of XPB, along with 17 residues of the N-terminal His_6_ tag, and residues 1 through 467 of Bax1.

### Purification, crystallization and structural determination of the StXPB–Bax1 complex

The expression plasmids pET15b/StXPB (Protein Accession number: WP_010979669) and pET15b/StBax1 (Protein Accession number: WP_010979670), generously provided by Dr Yulong Shen at Shandong University of China (2), were transformed into *E. coli* Rosetta (DE3) pLysS competent cells. Purification of His_6_-StXPB began with re-suspension of pelleted expression culture in lysis buffer (50 mM Tris–Cl pH 7.5, 500 mM NaCl, 0.01% (w/v) sodium azide). Cells were lysed by sonication and submitted to heat-denaturation and centrifugation to separate precipitated *E. coli* proteins from the thermo-stable recombinant protein. The soluble protein fraction was precipitated by addition of ammonium sulfate. After centrifugation, pelleted protein was re-solubilized in buffer A supplemented with 20 mM imidazole, loaded onto a HisTrap affinity column (GE), and eluted in buffer B. Peak fractions were combined and further purified by HiTrap SP ion exchange chromatography (GE). Protein eluted in buffer C (50 mM MES pH 6.0, 1000 mM NaCl, 0.01% (w/v) sodium azide) was concentrated and applied to a HiPrep 16/60 Sephacryl S-200 gel filtration column (GE) equilibrated in buffer D. His_6_-StBax1 purification was carried out as for StXPB, with the ion exchange step being omitted. HisTrap elution fractions of StBax1 were mixed with purified StXPB in a 1:1.2 molar ratio, then put through gel filtration in buffer D. Peak elution fractions containing the complex were concentrated to 20 mg/ml for crystallization experiments.

The StXPB–Bax1 complex was crystallized by vapor diffusion at room temperature in a 1:1 ratio of protein with reservoir solution (100 mM Tris–Cl pH 8.0, 50 mM sodium carbonate, 32% PEG-400). Crystals were harvested and flash frozen in a 100 K nitrogen stream, and stored for shipment to a synchrotron facility for data collection.

The StXPB–Bax1 complex diffraction data were collected at the Advanced Light Source (beamline 12.3.1, SIBYLS), Berkeley, CA. X-ray data processing and structure solution were carried out as for the AfXPB–Bax1 complex. The StXPB–Bax1 complex structure was solved by molecular replacement using the StXPB (PDB entry: 5TNU) N-terminal half (amino acids 1–229), C-terminal half (amino acids 240–439), and AfBax1 structure (PDB entry: 6P66) as search models. The asymmetric unit contains three copies of the StXPB-Bax1 heterodimer in the asymmetric unit encompassing amino acid residues 1 through 439 for XPB, with up to an additional 5 amino acids of the 6xHis-tag with observable electron density, and from amino acid residue 1 up to 481 of Bax1, thus encompassing the entire Bax1 sequence.

### Isothermal titration calorimetry (ITC) analysis

ITC measurements were performed using a MicroCal iTC200 calorimeter (GE). Titrations were performed in Af-protein sample buffer (10 mM Tris–Cl pH 7.5, 500 mM NaCl, 5% (v/v) glycerol, 0.01% (w/v) sodium azide) and St-protein sample buffer (10 mM Tris–Cl pH 7.5, 200 mM NaCl, 5% (v/v) glycerol, 0.01% (w/v) sodium azide). Protein sample concentrations were verified by absorbance at 280 nm. Purified samples of His_6_-AfXPB (139.5 μM) or His_6_-AfXPB-CTD (202 μM), and His_6_-StXPB (30 μM) were injected into His_6_-AfBax1 (13.3 μM) and His_6_-StBax1 (3 μM), respectively, with 16 injections at 2.36 μl per injection at a constant temperature of 40°C to inhibit precipitation. Similar procedures were applied to ITC titrations for mutants. Heats of dilution were subtracted from the raw data. All injections fit the single binding site mechanism with 1:1 stoichiometry and were repeated three times. The values for the stoichiometry of binding (*N*), enthalpy change (Δ*H*), and binding constant (*K*_b_) were determined via least squares analysis performed by the ORIGIN software package provided by the calorimeter manufacturer (GE) following the procedure provided by the manufacturer. The values for the change in free energy (Δ*G*_b_) and the change in entropy (Δ*S*) were then calculated as

$$\begin{equation*}{\Delta {G_{\rm b}}} = -RT*{\rm In}({K_{\rm b}})={\Delta H-T \Delta S}\end{equation*}$$

where *R* denotes the gas constant and *T* is the absolute temperature. Equilibrium association constants (*K*_b_) was also expressed as an equilibrium dissociation constant *K*_d_ = 1/*K*_b_.

### ATPase activity assay

ATPase reactions were carried out in the ATPase buffer (50 mM HEPES, pH 8.2, 100 mM KCl, 5 mM MgCl_2_, 1 mM DTT) with 1 mM ATP in a 50°C water bath. Protein and DNA sample concentrations were verified by *A*_280_ and *A*_260_, respectively. The concentration of liberated phosphate from hydrolyzed nucleotides was detected using published protocols (44). The absorbance of reactions with nucleotide alone was subtracted from protein reactions to account for ATP auto-hydrolysis.

### Nuclease activity assay

#### DNA substrate preparation

DNA oligonucleotides were ordered from IDT and purified from urea denaturing PAGE gel. Then ssDNA was 5′ end labeled for 30 min at 37°C in a 20-μl reaction containing 250 nM ssDNA, 0.625 μCi/μl [γ-32P] ATP, 1× T4 polynucleotide kinase reaction buffer, and 25 units of T4 polynucleotide kinase (Promega). Labeled ssDNA was subsequently annealed with the complementary ssDNA in a ratio of 1:1.2 in a buffer containing 20 mM Tris, pH 7.5; 50 mM NaCl; 1 mM EDTA. B50 and TAG31 were labeled. 50–16 bubbled DNA were annealed from labeled B50 and B50bub16. DNA oligo sequences are listed below:

B50: 5′-CCT CGA GGG ATC CGT CCT AGC AAG CCG CTG CTA CCG GAA GCT TCT GGA CC-3′

B50bub16: 5′-GGT CCA GAA GCT TCC GGA TAG TTA CCG CAC GAT GGA CGG ATC CCT CGA GG-3′

#### Nuclease assay

Reactions were incubated for 1hr at 48°C on a heat block in a total volume of 7 μl containing 20 mM Tris, pH 8.0; 40 mM NaCl; 10% glycerol; 0.1 mg/ml BSA; 10 mM MgCl_2_. Each reaction contained ∼100 fmol labeled DNA substrate and ∼20 000 fmol proteins or as indicated in the text. 3 μl stop buffer containing 90% formamide and 10 mM EDTA was added and reaction mixtures were boiled for 10 min prior to electrophoresis at 1500 V in a 18% urea polyacrylamide gel. Gels were exposed to a phosphorimaging screen overnight, visualized by a GE Typhoon 9410 Molecular Imager and edited by the Image Lab software.

## RESULTS

### Bax1 interacts exclusively with the C-terminal half of XPB in solution

**Figure 1.**
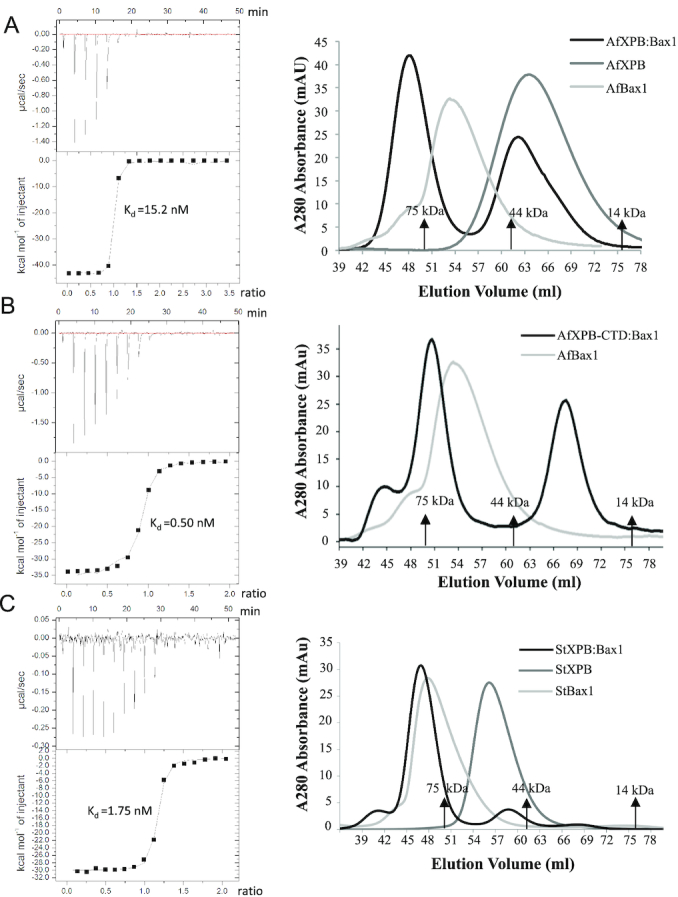
The interactions of XPB with Bax1. (**A**) The interaction of AfXPB with AfBax1. (**B**) The interaction of AfXPB-CTD with AfBax1. (**C**) the interaction of StXPB with StBax1. Left: profiles of ITC titrations; Each titration represents a typical profile of multiple assays with the raw data in the top and data fitting by the ORIGIN software (GE Healthcare) in the bottom. right: profiles of S200 size-exclusion chromatography. The positions of three protein markers are indicated based on chromatographic calibration profile (Supplementary Figure S1) of Conalbumin (75 kDa), Ovalbumin (44 kDa) and Lactalbumin (14 kDa).

Bax1 has been reported to form a heterodimer with XPB from several archaea including *T. acidophilum* (38,40), *S. solfataricus* (39) and *S. tokodaii* (2). Furthermore, *S. solfataricus* XPB and *S. acidocaldarius* Bax1 were reported to form a cross species heterodimer (37). However, it is yet far from clear how they interact with each other. We first characterized the interaction by isothermal titration calorimetry (ITC) using purified recombinant XPB and Bax1 proteins. AfXPB forms a heterodimer with AfBax1 at a *K*_d_ of 15 nM while StXPB forms a heterodimer with StBax1 with almost 10 times higher affinity (*K*_d_ of 1.75 nM) (Figure 1). Interestingly, the C-terminal half (residues 234–452) of AfXPB is sufficient for the formation of the XPB–Bax1 complex and has 30 times higher affinity (*K*_d_ of 0.50 nM) (Figure 1B). Size-exclusion chromatography results (Figure 1) show both AfXPB and StXPB are monomers in solution. However, StXPB (at 55 ml peak, Figure 1C) and AfXPB (at 65 ml peak, Figure 1A) were eluted differently even if they are very close in molecular weight (51 kDa for StXPB and 50 kDa for AfXPB). AfXPB was eluted much slower than expected, possibly due to its open conformation as observed in the crystal structure (1). To our surprise, StBax1 forms exclusively homodimers (eluted at 44 ml peak, Figure 1C) in solution while AfBax1 primarily exists as a monomer (eluted at 54 ml peak, Figure 1A) with a fraction of homodimers (eluted at 48 ml shoulder, Figure 1A). However, both AfBax1 and StBax1 form the XPB–Bax1 heterodimer exclusively with their full length XPB partner (Figure 1).

### Crystal structure of the AfXPB–Bax1 complex

**Figure 2.**
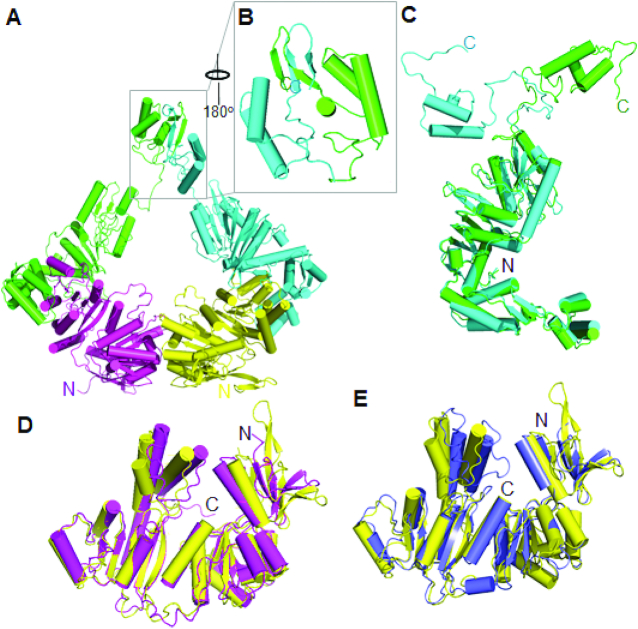
The two AfXPB–Bax1 heterodimers in the asymmetric unit. (**A**) Ribbon presentation of the two AfXPB–Bax1 heterodimers. AfXPB molecules are colored in yellow and magenta, AfBax1 molecules are colored in cyan and green. (**B**) The zoom-in view of the C-termini of AfBax1. (**C**) Structural comparison of the two AfBax1 molecules. (**D**) Structural comparison of the two AfXPB molecules. (**E**) Structural comparison of AfXPB in the heterodimer (yellow) and free of Bax1 (blue, PDB ID 2FWR, chain D) (1).

To further characterize the interactions of XPB with Bax1 and understand why the C-terminal half AfXPB binds much more strongly to AfBax1 than the full length AfXPB, we determined the crystal structure of the AfXPB–Bax1 complex using recombinant proteins expressed and purified from *E. coli* culture. The crystal structure was determined to 3.0 Å resolution by molecular replacement combined with anomalous data obtained from an isomorphous crystal of AfXPB complexed with a Se-Met derivative AfBax1 (Supplementary Table S1). There are two copies of the AfXPB–Bax1 heterodimer in the asymmetry unit forming a heart-shape (Figure 2A). The two AfBax1 molecules interact with each other through the C-termini, particularly, the β-strand loop β-strand (βLβ) C-terminal tails forming two pairs of anti-paralleled β-strands (Figure 2B). The two AfBax1 molecules have almost the same structure except the C-terminal domain oriented differently for dimerization (Figure 2C), indicating that the C-terminal domain is flexibly attached to the rest of Bax1 allowing different positions for protein-protein interactions. The two AfXPB molecules remain the open conformation observed in the AfXPB crystal structure (1) (Figure 2D and E). This open conformation does not form the ATP binding groove between the two helicase domains (HD1/2) unless it turns into the closed conformation by domain rotation (1) (see Supplementary movie). Although the two AfXPB molecules have direct contacts with each other through HD1 in the crystals (Figure 2A), we did not observe any AfXPB dimer in solution (Figure 1A). However, we did observe a fraction of AfBax1 dimer in solution by size-exclusion chromatography as described in the above (Figure 1B). The AfXPB–Bax1 complex structure reveals that AfBax1 exclusively interacts with the C-terminal half XPB including the ThM and HD2 (Figure 2A). This is consistent with the size-exclusion chromatography and ITC results (Figure 1B): AfBax1 formed a stable complex with the AfXPB C-terminal half. Taken together, when AfBax1 interacts with the full length AfXPB, AfBax1 dimerization brings the two AfXPB partners together. However, the interactions between the AfXPB molecules destabilize the AfBax1 dimerization as the *K*_d_ for the full length AfXPB binding with AfBax1 is 15.2 nM, about 30× weaker than the *K*_d_ of 0.50 nM for the C-terminal half AfXPB binding with AfBax1 (Figure 1). This is likely due to the same electrostatic potential surfaces of the N-termini between the two AfXPB molecules (Supplementary Figure S2), which produce repelling forces to push away each other to destabilize the dimerization of the XPB–Bax1 complexes in solution. Therefore, both the AfBax1 dimer (the shoulder eluted at 48 ml in Figure 1A) and monomer (peak at 54 ml in Figure 1A) interact with AfXPB to form the AfXPB–Bax1 heterodimer (peak at 48 ml in Figure 1A) exclusively.

**Figure 3.**
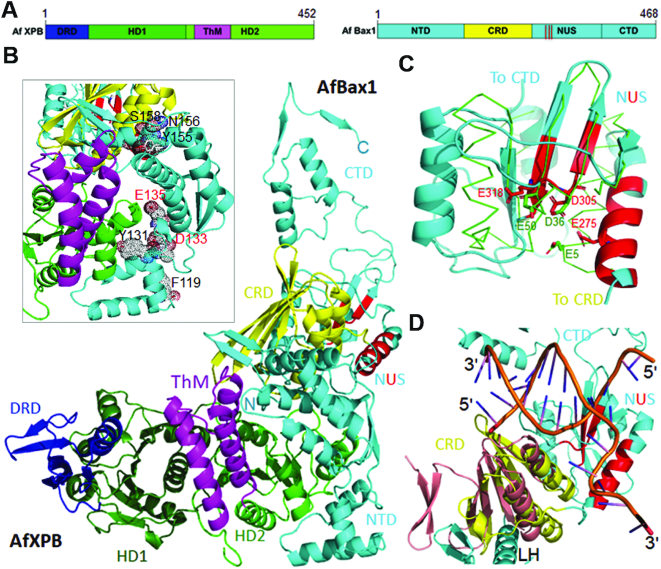
Structure of the AfXPB–Bax1 complex. (**A**) Diagrams of domain arrangements in AfXPB and AfBax1. Domains are presented as boxes in different colors with labels: DRD (damage recognition domain), HD1 (helicase domain 1), HD2 (helicase domain 2) and ThM (thumb-like) domains of AfXPB are colored in blue, dark green, green, and magenta; NTD, CRD, NUS, and CTD of AfBax1 are colored in cyan, yellow, cyan with red lines for three conserved nuclease motifs, and cyan. (**B**) The crystal structure of the AfXPB–Bax1 complex in cartoon. Insertion: the interface between AfXPB and Bax1. Residues equivalent to those mutated in ref. (40) are shown as dot spheres with labels. Two acidic residues for the potential N-terminal nuclease active site are indicated by red labels. (**C**) Superimposition of VRR-Nuc domain (46) (PDB entry: 4QBN, in green ribbons) over the NUS domain of AfBax1. Active site residues are shown in sticks. (**D**) Overlay of Cas2-forked DNA (PDB entry: 5DQU) (48) on the CRD of AfBax1. Cas2 is colored in wheat and DNA in brown.

The crystal structure of the AfXPB–Bax1 complex reveals that AfBax1 consists of four domains (Figure 3): the N-terminal domain (NTD), a central domain (CRD), the nuclease (NUS) domain and the C-terminal domain (CTD). The NTD (residues 1-146) starts with a β-hairpin (β1TTβ2, Supplementary Figure S3), followed by a tandem of tri-helix bundles (α1–3 and α4–6) and a β-strand (β3 and β4). The second β-strand (β4) loops back to form an anti-parallel pair with the first β-strand (β3) connecting the two tri-helix bundles. The NTD is connected to the CRD through a long α-helix (LH in Figure 3D and α7 in Supplementary Figure S3). The NTD is mainly responsible for the interactions with XPB: the β-hairpin and the first tri-helix bundle (α1–3) interact with the ThM domain of XPB while the second tri-helix bundle (α4–6) interacts with the HD2 of XPB (Figure 3B). These interactions seem important to the activity of DNA incision by the XPB–Bax1 complex since mutations on residues in these interaction interfaces (Figure 3B, insertion) have been shown previously to reduce significantly the nuclease activity of the XPB–Bax1 complex (38).

Archaeal Bax1 was originally identified by a combination of sequence homology and secondary structural analysis as a nuclease involved in DNA repair, which contains three conserved motifs of the DUF790 nuclease family (45). The nuclease (NUS) domain of Bax1 (residues 270–372, Figure 3C and Supplementary Figure S3) consists of a five mixed β-strands sheet (β10–14) flanked by two α-helices on each side (Figure 3D), which shows structural similarity with a viral type replication and repair nuclease (VRR-Nuc) (Dali Z-score of 4.0, corresponding to 3.6 Å r.m.s.d. over 103 residues (270–372)). VRR-Nuc is a member of the ancient restriction endonuclease-like superfamily with a mixed α/β fold of αβββαβ topology (45). VRR-Nuc containing proteins usually exist as a single domain nuclease in many bacteria and viruses. FANCD2/FANCI-associated nuclease 1 (FAN1) is the only example of multi-domain eukaryotic protein containing a VRR-Nuc domain. FAN1 is a structure specific nuclease required for the repair of inter-strand DNA crosslinks like the ERCC1-XPF nuclease (46). When the structure of *Salmonella phage SETP3* VRR-Nuc domain (PDB entry: 4QBN, green wires in Figure 3C) is superimposed with the nuclease domain of Bax1 (cyan ribbons in Figure 3C), the three acidic residues from the conserved motifs of Bax1 (E275, D305 and E318) align well with the three acidic residues (E5, D36 and E50) at the active site of the VRR-Nuc (46). These three acidic residues (E275, D305 and E318) potentially form the metal binding site at the Bax1 nuclease active site and locate near the phosphate backbone of the 5′-overhang strand (Figure 3, C and D), therefore allowing Bax1 to cut the DNA strand 3′ to the damage (at the center of the bubble), strongly supporting its biochemical activity as an XPG-like nuclease (39). The NUS domain is connected through a long loop to the CTD (residues 400–468, Figure 3B and Supplementary Figure S3), which is likely a domain for protein-protein interactions including self-dimerization as observed in the crystal (Figure 2, A and B).

The CRD (residues 168–260, Figure 3D) consists of a five anti-paralleled β-strands (β4–9 in Supplementary Figure S3) sheet with three α-helices (α8–10 in Supplementary Figure S3) on one side. Structural homology search by the Dali server (47) suggests that the CRD shares structural similarity with casp8-associated protein 2 (Cas2, PDB entry: 5DQU, E chain) (Dali Z-score of 5.6, corresponding to 2.9 Å r.m.s.d. over 87 residues (168–255)). When we superimposed Cas2 from the crystal structure of *E. coli* Cas1-Cas2 bound to a forked DNA (PDB entry: 5DQU) (48), Cas2 (brown color in Figure 3D) matches well with the CRD (yellow color in Figure 3D) of AfBax1. In addition, the N-terminal β-hairpin (β1 and β2, cyan color) of Bax1 provides an extra element to match the two additional β-strands in Cas2. Interestingly, the structural alignment places the forked DNA right at the nuclease domain of Bax1 with the conserved nuclease motifs (red highlights in the NUS domain, Figure 3) near the ds–ssDNA junction, in agreement with the role of Bax1 as an XPG-like nuclease in DNA incision.

### AfBax1 contains two distinguished nuclease active sites

**Figure 4.**
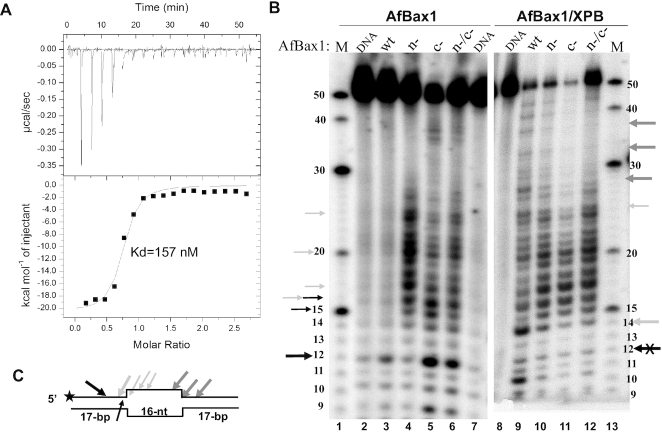
AfBax1 contains two distinguished nuclease active sites in the N-terminal domain and the nuclease domain. (**A**) The N-terminal active site plays a role in Bax1-XPB interactions. Mutation D133A/E135A increases the *K*_d_ of the AfXPB–Bax1 complex by 10-fold. ITC titration represents a typical profile of multiple assays with the raw data in the top and data fitting by the ORIGIN software (GE Healthcare) in the bottom. (**B**) DNA incisions on a 16-nt bubble DNA substrate by AfBax1 variants and their complexes with AfXPB. M: DNA oligomer markers, DNA: nuclease reaction control without AfBax1 or AfBax1-XPB complex, wt: wild type AfBax1, n-: AfBax1 mutant D133A/E135A, c-: AfBax1 mutant D305A, n-/c-: AfBax1 mutant D133A/E135A/D305A. Black arrows indicate incised products by the N-terminal nuclease active site (X indicates inhibition on the activity); Grey and light gray arrows indicate incised products by the nuclease domain. (**C**) Schematic summary of the results from (B). The star indicates *P*-32 label on the DNA strand.

Previously euryarchaeal TaBax1 was reported to cleave 3′-overhang DNA substrates at 4–6 bases away from the junction in the single-stranded tail (38) while crenarchaeal SsBax1 has no such activity by itself but works together with SsXPB to cleave 5′-overhang at the junction (39), showing different strand selection than TaBax1. Furthermore, substitution of residues Phe116, Tyr128, Asp130, Glu132, Tyr152 and Asn153 with alanine significantly reduced the nuclease activity of TaBax1 (38), leading to the proposal that these residues form the nuclease active site of TaBax1. These residues correspond to Phe119, Tyr131, Asp133, Glu135, Tyr155 and Asn156 of AfBax1 at the interaction interface between XPB and Bax1 (Figure 3B and Supplementary Figure S3), away from the conserved nuclease domain where substitution of SsBax1 residue Asp-301, a key acidic residue at the conserved nuclease domain (Asp305 in Figure 3C and Supplementary Figure S3), with alanine eliminated the nuclease activity of the SsXPB–Bax1 complex (39). These results suggest that there are two nuclease active sites in Bax1 nuclease and protein-protein interactions regulate the polarity of DNA incision by the Bax1 nuclease and the Bax1-XPB complex in order to remove a fragment of damage DNA during DNA repair. To test this hypothesis, we substituted acidic residues Asp133 and Glu135 with alanine at the N-terminal AfBax1 and acidic residue Asp305 with alanine at the nuclease domain of AfBax1, and tested their influences on protein-protein interactions and nuclease activities (Figure 4). AfBax1 with substitutions D133A/E135A still interacted with AfXPB but with much lower affinity (*K*_d_ = 157 nM in Figure 4A) comparing to the wild type AfBax1 (*K*_d_ = 15.2 nM in Figure 1A) based on ITC measurements, conforming the importance of these residues in protein-protein interactions as observed in the crystal structure (Figure 3B, insertion).

Nuclease activity assays with a 50-bp long substrate containing a 16-nt bubble (Figure 4B and C) reveal that AfBax1 has two nuclease active sites with distinguished nuclease activities. AfBax1 alone shows weak nuclease activity by making an incision (indicated by the black arrow in Figure 4C) at the 5′ ds region with 5-bp away from the ds-ss junction to produce a 12-nt product (compare lane 3 with lane 2 in Figure 4B). The N-terminal nuclease site is likely responsible for this activity since mutation D133A/E135A almost eliminated this activity (compare lane 4 to lane 3 in Figure 4B). However, inhibition on the N-terminal nuclease activity by the D133A/E135A mutation enhanced the nuclease activity from the nuclease domain, which incises DNA around the ds-ss junction to produce products (indicated by slim light grey arrows in Figure 4B and C) with various sizes ranging from 14-nt to 26-nt (lane 4 in Figure 4B and C). Mutation D305A in the nuclease domain significantly reduced this new activity (lane 5 in Figure 4B) but enhanced the N-terminal nuclease activity as revealed by increased level of the 12-nt product (indicated by the black arrow in Figure 4B). In addition, the increased levels of the15-nt and 16-nt products for the AfBax1 mutant D133A/E135A suggest that the N-terminal nuclease active site can perform DNA incision at the ds-ss junction (indicated by slim black arrow in Figure 4C) as well. These results demonstrated that the N-terminal nuclease active site competes with the nuclease domain for DNA incision, and inhibition on either activity significantly enhances the other activity. Therefore, the AfBax1 mutant D133A/E135A/D305A (lane 6 in Figure 4B) displayed stronger activities for both the N-terminal nuclease and the nuclease domain than the wild type AfBax1 (lane 3 in Figure 4B) because the inhibition caused by mutation is overcome by the enhancement from the other active site.

As being expected, the incision by the N-terminal nuclease active site to produce the 12-nt product was inhibited for the AfXPB–Bax1 complex (compare lane 9 with lane 3 in Figure 4B) because the interaction of AfXPB with AfBax1 likely blocks DNA from accessing the N-terminal nuclease active site of AfBax1. This leads to the enhancement on the activity from the nuclease domain to produce products longer than 12-nt (lane 9 in Figure 4B). Interestingly, the AfXPB–Bax1 complex displayed the ability to incise DNA at the other ds-ss junction of the 16-nt bubble (indicated by grey arrows in Figure 4C) to produce products of 28-nt, 32-nt, and 37-nt DNA oligomers (lane 9 in Figure 4B). As shown in Figure 3D, the CRD interacts with forked DNA like Cas2 to allow the nuclease domain of AfBax1 to incise the 5′ arm at the ds-ss junction. These products were significantly reduced by the mutation D133A/E135A in AfBax1 (lane 10 in Figure 4B) and were almost eliminated by the mutation D305A in AfBax1 (lane 11 in Figure 4B). These results indicated that the incision at the other ds-ss junction of the bubble is mediated by the nuclease domain of AfBax1 but is regulated by the interactions between AfXPB and AfBax1 since the AfBax1 mutant D133A/E135A interacts with AfXPB much weaker (*K*_d_ = 157 nM) than the wild type AfBax1 (*K*_d_ = 15.2 nM).

### Structure of the StXPB–Bax1 complex

**Figure 5.**
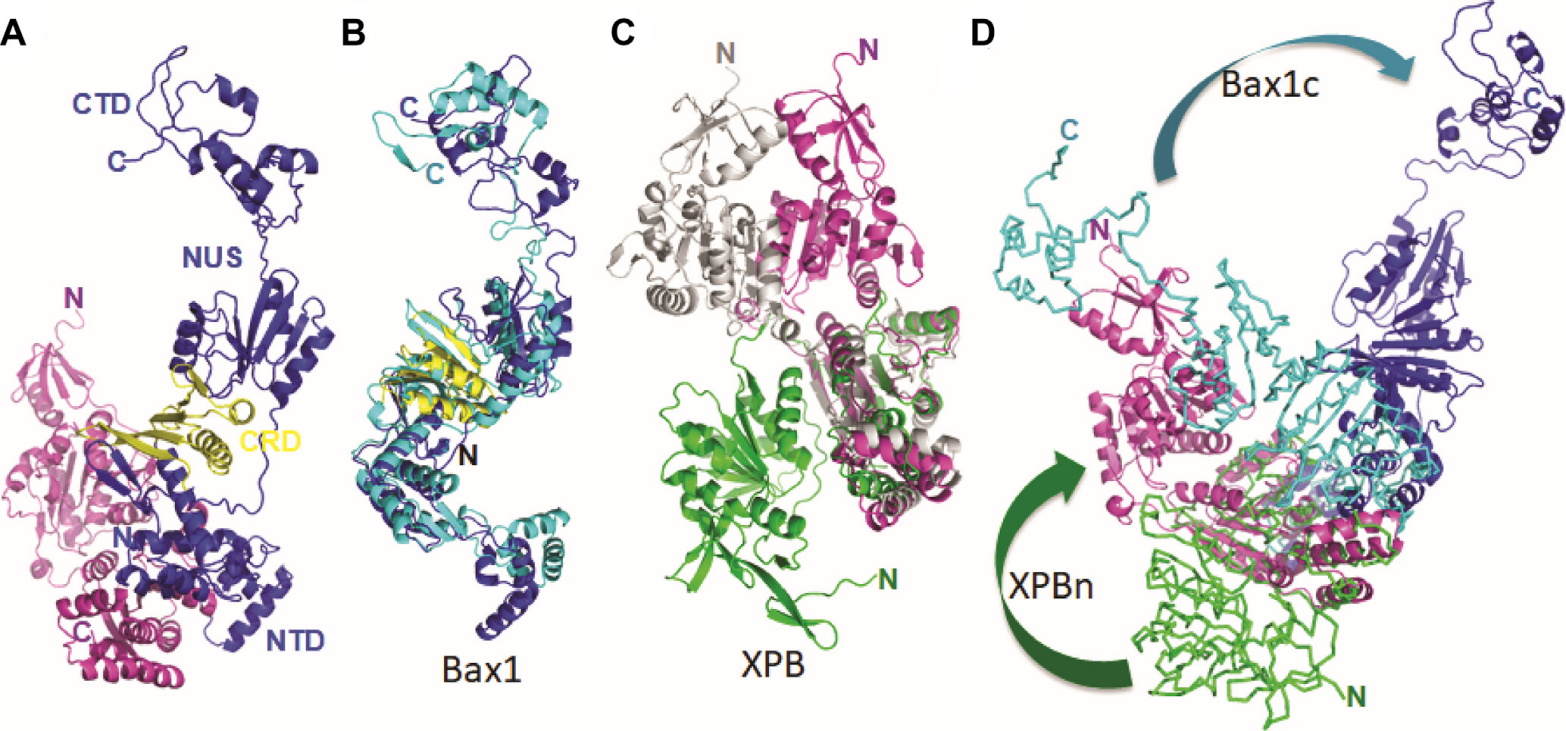
The XPB–Bax1 complex is a dynamic machine. (**A**) Crystal structure of the StXPB–Bax1 complex in cartoon. StXPB is colored in magenta. StBax1 is colored in blue with CRD in yellow. (**B**) Superimposition of AfBax1 (in cyan cartoon) with StBax1 (as in A). (**C**) Superimposition of StXPB from the heterodimer structure (as in A) with the StXPB crystal structure (32) (PDB entry: 5TNU, chain A in gray cartoon) and the AfXPB crystal structure (1) (PDB entry: 2FWR, chain D in green cartoon) over the HD2 and ThM domains. The chain B of StXPB from 5TNU is in the same conformation as StXPB from the heterodimeric structure but is not shown for clear visibility. (**D**) Structural comparison of the StXPB–Bax1 complex with the AfXPB–Bax1 complex. The AfXPB–Bax1 complex is superimposed with the StXPB–Bax1 complex over the HD2 of AfXPB and StXPB. The AfXPB–Bax1 complex is displayed in ribbons with AfXPB in green and AfBax1 in cyan. Different orientations of the N-terminal half XPB (XPBn) and the C-terminal half Bax1 (Bax1c) between the two heterodimers are highlighted by arrows.

We previously observed that AfXPB is in the open conformation (1) but StXPB is in two partially closed conformations (32), suggesting differences in archaeal species. In addition, Bax1 from *T. acidophilum* (38) behaves differently than Bax1 from *S. solfataricus* (39). In order to see structural differences in the XPB–Bax1 complex from different archaea, we then determined the crystal structure of the StXPB–Bax1 complex up to 3.15 Å resolution (Figure 5A, statistics in Supplementary Table S1). The crystal structure indicates that StBax1 is very similar to that of AfBax1 consisting of four domains (Figure 5B). Each domain except the C-terminal domain has the similar fold like those in the AfBax1 structure (Figure 5B and Supplementary Figure S3). The C-terminal domain of Bax1 is least conserved among archaeal Bax1 homologs even in amino acid sequence and is absence in many archaeal Bax1 (Supplementary Figure S3).

There are three StXPB–Bax1 dimers forming a triangle in the asymmetric unit of the crystal (Supplementary Figure S4). The three StXPB molecules have the same conformation, so do the three StBax1 molecules. Each angle of the triangle is formed by the interface of the StXPB–Bax1 heterodimer plus the C-terminal domain of another Bax1 from a nearby dimer interacting with the second tri-helix bundle in the N-terminal domain of the first Bax1 (Supplementary Figure S4). Interestingly, StBax1 remains exclusively as a homodimer (Figure 1C, peak at 45 ml) in solution but interactions of StBax1 with StXPB completely eliminate StBax1 homodimers, suggesting that the C-terminal half of StXPB competes with the C-terminal domain of StBax1 to interact with the N-terminal domain of StBax1 for dimerization.

**Figure 6.**
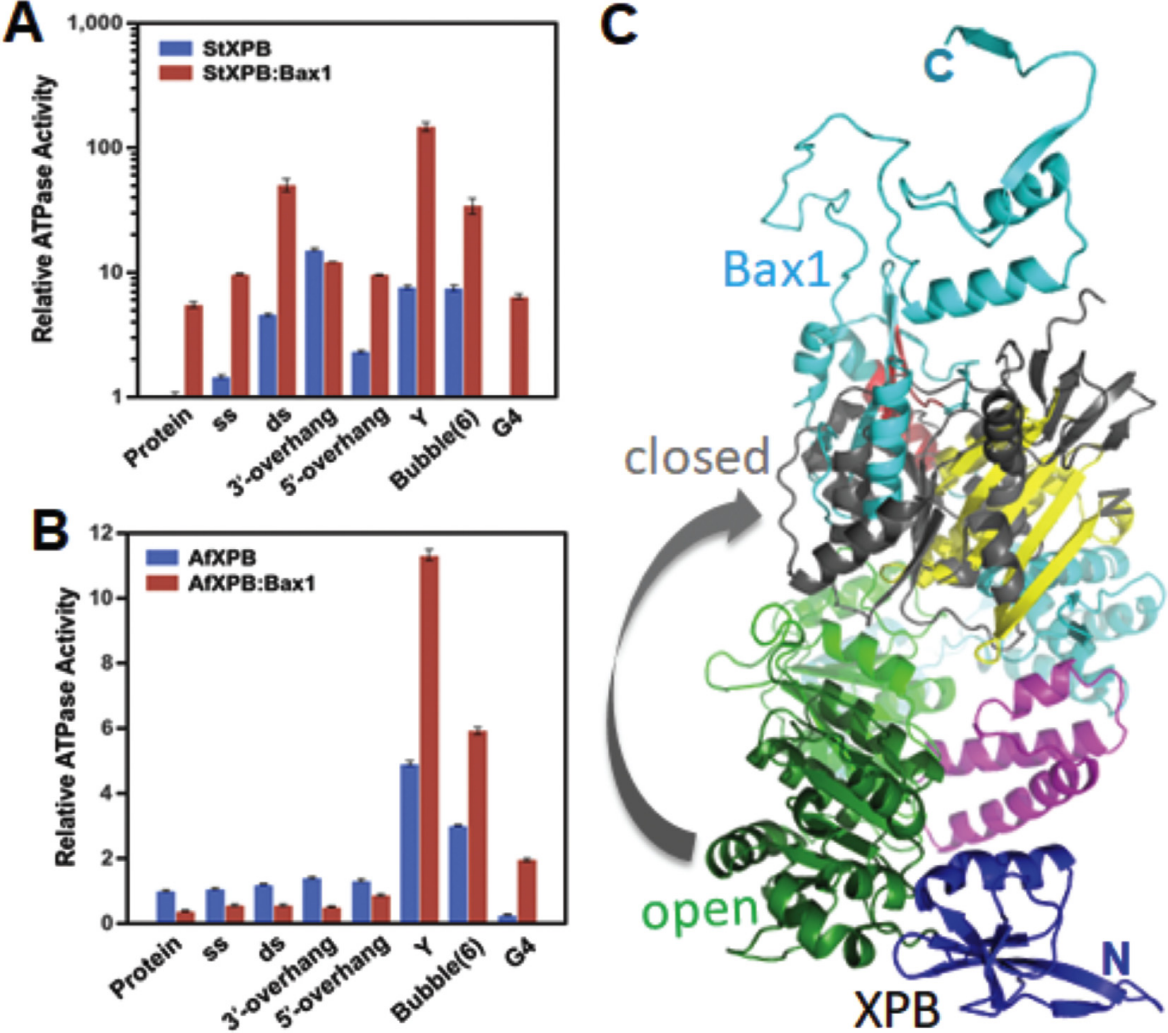
The impact of various DNA substrates on the ATPase activity of XPB and the XPB–Bax1 complex. ATPase activities of *A. fulgidus* (**A**) or *S. tokodaii* (**B**) XPB or the XPB–Bax1 heterodimer were assayed in the absence or presence of a 1:1 molar ratio of protein to DNA for various DNA substrates. ss: single stranded oligonucleotide, 5′-GCCGTGCGCATTCGCCGTGTGGAGCCTGTC-3′; ds: double stranded oligonucleotide, 5′-TGACTCAACATGGAAACCTACAAT-3′; 3′-overhang: -5′-CGAGCACTGCAGTGCTCGTTGTTAT-3′, 3′-GCTCGTGACGTCACGAGC-5′; 5′-overhang: 5′-TATTGTTCGAGCACTGCAGTGCTCG-3′, 3′-GCTCGTGACGTCACGAGC-5′; Y: forked oligonucleotide, 5′-GACAGGCTCACACGTTACGTTGCGCACGGC-3′, 3′-AAAAAAATTCCCGCAATGCAACGCGTGCCG-5′ bubble(6): double stranded oligonucleotide with a 6-bp mismatched bubble in the middle, 5′-TTGACTCAACATCCTTTGCTACAATCAGT-3′, 3′-AACTGAGTTGTATTTCCAGATGTTAGTCA-5′; G4: G-quadruplex oligonucleotide, 5′-TGGACCAGACCTAGCAGCTATGGGGGAGCTGGGGAAGGTGGGAATGTGA-3′; Base-paired nucleotides are underlined. The ATPase activity of StXPB (5.0 μM [Phosphate]/μM protein per minute) and AfXPB (0.4 μM [Phosphate]/μM protein per minute) is used as the base activity in (A) and (B), respectively. The standard deviations are calculated from at least three measurements of the same reaction. (**C**). AfBax1 hinders the formation of the closed AfXPB conformation. The crystal structure of the AfXPB–Bax1 heterodimer (as in Figure 3A) is superimposed with the closed AfXPB conformation model (1). The N-terminal (DRD and HD1) AfXPB in the closed model is in gray cartoon.

In the StXPB–Bax1 complex (Figure 5A), the N-terminal domain of StBax1 interacts with the C-terminal half of StXPB similarly to that in the AfXPB–Bax1 complex. However, StXPB is in a partially closed conformation similar to one of the conformations (32) observed in the absence of Bax1 (Figure 5C). StBax1 rotates away to avoid clashing with the N-terminal half of StXPB compared to the AfXPB–Bax1 complex (Figure 5D). The StXPB–Bax1 complex keeps StXPB in the more closed conformation (Figure 5C) as observed previously in the StXPB crystal structure (*32*), leading to 5× stimulation on the ATPase activity of StXPB (Figure 6A). In contrast, AfBax1 reduces the ATPase activity of AfXPB by 50% (Figure 6B) instead because the AfXPB–Bax1 heterodimer keeps AfXPB in the open conformation and hinder AfXPB from forming the closed conformation required for ATP binding as the C-terminal half of AfXPB collides with the CRD and NUS domains of AfBax1 when the closed conformation of AfXPB is docked onto the complex structure (Figure 6C).

At the molecular level, the XPB–Bax1 complex is built to be a dynamic machinery to fulfill its biological functions for DNA unwinding and damage removal. In both the AfXPB–Bax1 and StXPB–Bax1 structures, the XPB–Bax1 interface is made of the interactions between the helices of the ThM domain of XPB with the first tri-helix bundle of Bax1 as well as the interactions between the helices of the HD2 of XPB with the second tri-helix bundle of Bax1. These helix-helix interactions provide the flexibility to allow Bax1 to swing back and forth while the flexible link between the HD1 and HD2 of XPB allows the N-terminal half XPB to rotate from open conformation to closed conformation (Figure 5D and the Supplementary movie). As shown in Figure 6, interactions with Y-shaped DNA and bubbled DNA substrates induce the XPB–Bax1 complex to change conformations resulting in about 10× stimulation of their ATPase activity. Through this dynamic change, the AfXPB–Bax1 complex overcomes the inhibition by AfBax1 and has 2× higher ATPase activity than AfXPB alone (Figure 6B).

**Figure 7.**
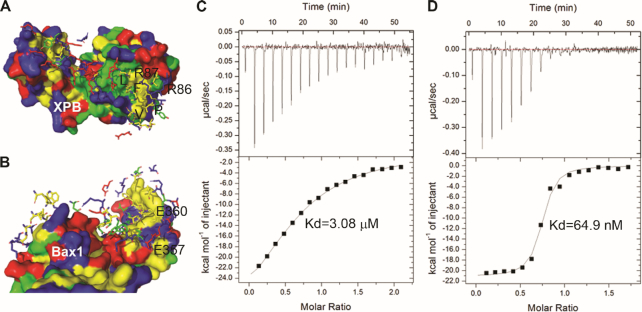
Both hydrophobic and charge/polar interactions contribute to the interactions of XPB with Bax1. (**A**) Biochemical properties of the StXPB surface interacting residues from StBax1: E24, D27, E31, K41, G43, E44, D45, E47, E48, E50YLEKIY56, R62, I83, R86, R87, L89 (label L), F90 (label F), K91YG93, P94 (label P), V95 (label V), L96, E98, R101, I104, I105, M117, V120, F121 and D123LDEE127. Residues selected for mutagenesis are labeled. (**B**) Biochemical properties of StBax1 surface interacting with residues of StXPB: F278, V282, A285AK287, K289, R292, L295, L296, W298, H299, N303, R316, L319, K323, R332DTQ335, Y338, S341KTFLIPV348, T350YKTD354, E357, E360, I361, K364, E369YRV372, V378 and F379. Residues are represented by sticks and colored in the same way with the partner surface according to amino acid properties: yellow for hydrophobic residues, green for polar uncharged residues, red for acidic residues, and blue for basic residues. Biochemical characteristics of amino acid residues at the Bax1:XPB interface were determined by the PISA server (http://www.ebi.ac.uk/pdbe/prot_int/pistart.html). (**C**) ITC results for the interaction of StXPB with StBax1 mutant L89A/F90A/P94A/V95A. (**D**) ITC results for the interaction of StXPB mutant E357A/E360A with StBax1 mutant R86A/R87A. Each ITC titration represents a typical profile of multiple assays with the raw data in the top and data fitting by the ORIGIN software (GE Healthcare) in the bottom.

Both hydrophobic (van der Waals) and polar/charge interactions contribute to the assembly of the XPB–Bax1 complex (Figure 7A and B). We tested the impact of some of these residues by mutagenesis and ITC assays (Figure 7C and D). Compared to the interactions between the wild type StXPB and StBax1 (*K*_d_ = 1.75 nM, Figure 1C), StXPB mutant E357A/E360A interacted with StBax1 mutant R86A/R87A in a much weaker fashion (*K*_d_ = 64.9 nM) while the interactions of the StBax1 mutant L89A/F90A/P94S/V95S with the wild type StXPB have the highest *K*_d_ of 3.08 μM under the same condition. The interaction interfaces between XPB and Bax1 are conserved among archaea (Supplementary Figure S5).

## DISCUSSION

Here, we reported the studies on the interactions of XPB with Bax1 from one euryarchaeal *A. fulgidus* and one crenarchaeal *S. tokodaii*. We observed that AfBax1 is primarily a monomer in solution with a fraction of homodimer by size-exclusion chromatography, and AfBax1 forms a heterodimer with AfXPB, which breaks the AfBax1 homodimer (Figure 1). Interestingly, almost identical results were reported previously for euryarchaeal TaBax1 and TaXPB by size-exclusion chromatography and analytic ultracentrifugation (38). In contrast, the StBax1 is exclusively a homodimer in solution but interactions with StXPB also break the StBax1 homodimers into StXPB–Bax1 heterodimers. It seems that crenarchaeal SsBax1 is also a homodimer in solution since SsBax1 alone behaved similarly to the SsBax1-SsXPB heterodimer during size-exclusion chromatography (37). These results demonstrate the similarities and diversities between euryarchaeal and crenarchaeal Bax1/XPB proteins. The diversities were also reflected on their biochemical properties. AfBax1 reduces the ATPase activity of AfXPB to less than 50% while StBax1 increases the ATPase activity of StXPB by about 5 times (Figure 6). This difference can be explained by the structural differences between the AfXPB–Bax1 complex and the StXPB–Bax1 complex. In the absence of Bax1, AfXPB is in the open conformation while StXPB is in partially closed conformations as observed in the AfXPB (PDB entry: 2FWR) (1) and StXPB (PDB entry: 5TNU) (32) crystal structures, respectively. In the crystal structure of the AfXPB–Bax1 complex, AfXPB remains in the open conformation just like AfXPB alone with loss of the ATP binding groove. In order to form the ATP-binding groove, the N-terminal half (DRD and HD1) of AfXPB has to rotate about 170^o^ to form the closed conformation (see Supplementary movie). The presence of AfBax1 hinders this rotation as shown in Figure 6C, leading to the reduction on the ATPase activity of AfXPB. On the other hand, the StXPB–Bax1 complex keeps StXPB in a much more closed conformation (Figure 5C), which is favorable for ATP-binding. Therefore, StBax1 enhances significantly the ATPase activity of StXPB. Similar results were previously reported by others (2). The different XPB conformations (the open formation observed in both AfXPB and the AfXPB–Bax1 complex vs the closed conformation observed for StXPB) are not the results of crystal packing because AfXPB was crystallized in P1 space group while the AfXPB–Bax1 complex was crystallized in C2 space group, the same space group which the StXPB–Bax1 complex was crystallized in. Interestingly, the inhibitory effect of AfBax1 on the XPB ATPase activity is removed by Y-shaped or bubbled DNA substrates, which allow the AfXPB–Bax1 complex to have higher ATPase activity than AfXPB alone (Figure 5B), suggesting these NER intermediate DNA substrates activate the AfXPB–Bax1 complex, in agreement with its role in nucleotide excision repair. These results demonstrate that Bax1 can adjust its relative position to XPB allowing XPB to form a closed conformation or open conformation (see Supplementary movie), and this process is likely regulated by the interactions with DNA to coordinate DNA unwinding with incision during DNA repair.

**Figure 8.**
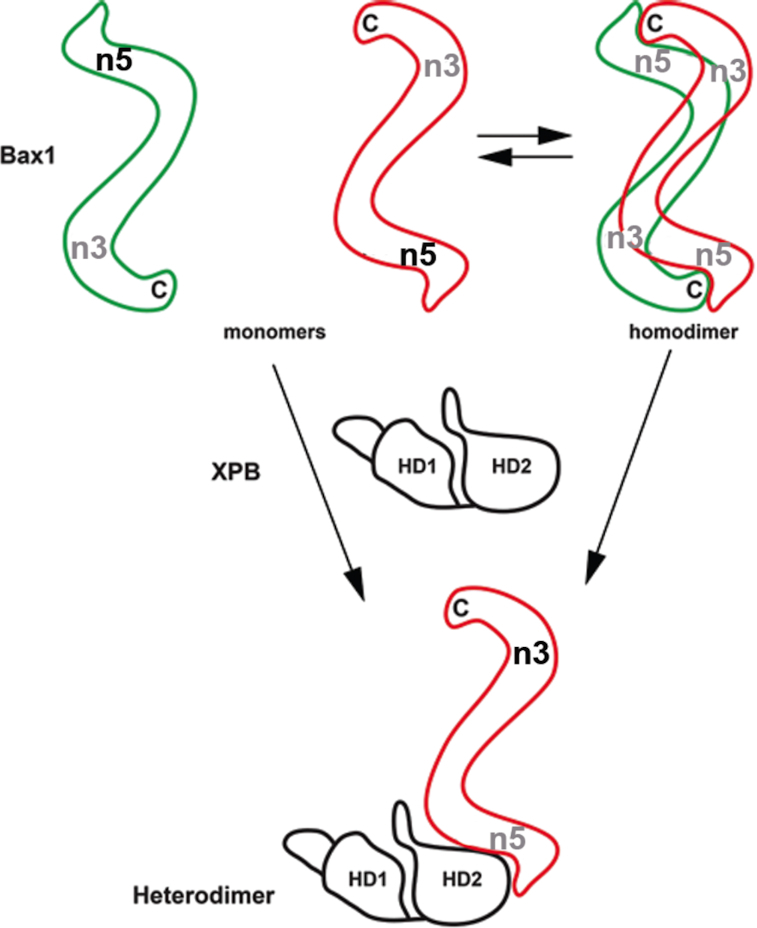
Protein-protein interactions regulate the nuclease activity of Bax1. Bax1 likely contains two nuclease active sites: one (n5) at the N-terminal domain and the other (n3) at the nuclease domain. Bax1 from euryacharchaea *A. fulgidus* and *T. acidophilum* is predominantly monomer in solution and is in the conformation preferable for the N-terminal (n5) activity, which presumably incises DNA 5′ to the damage in the middle of the bubble. StBax1 and SsBax1 from crenarchaea *Sulfurisphaera* are exclusively homodimers in solution and have no apparent nuclease activity (39) because the two nuclease active sites are mutually masked due to dimerization. Both monomeric and dimeric Bax1 interact with XPB to form the heterodimeric XPB–Bax1 complex which masks the N-terminal active site (n5) but enhances the activity of the nuclease domain (n3). The nuclease domain presumably incises DNA 3′ to the damage in the middle of the bubble. Active nuclease sites are highlighted by black labels (n3 or n5) while inhibited nuclease sites are labeled in gray. The C-terminus of Bax1 is indicated by letter C while the two helicase domains of XPB are labeled by HD1 and HD2.

Previously euryarchaeal TaBax1 was reported to cleave 3′-overhang DNA substrates at 4–6 bases away from the junction in the single-stranded tail (38) while crenarchaeal SsBax1 has no such activity by itself but works together with SsXPB to cleave 5′-overhang at the junction (39), showing different strand selection than TaBax1. Interestingly, nuclease activity assays with mutations of Bax1 suggest TaBax1 has a different active site than SsBax1 as well (38). Following these leads, we demonstrated here that AfBax1 contains two distinguished nuclease active sites. One nuclease active site is located in the N-terminal domain of Bax1 at the interaction interface between XPB and Bax1 (Figures 3 and 4), explaining why TaXPB inhibited the nuclease activity of TaBax1 (38) as the association of XPB will block this active site from access by any DNA substrates. The other is located in the conserved nuclease domain since Ala-substitution of Asp-301, a key acidic residue at the conserved nuclease domain previously identified by bioinformatic analysis (Figure 3C and Supplementary Figure S3), completely eliminated the nuclease activity of the SsXPB–Bax1 complex (39). Interestingly, we observed that inhibition of one nuclease site will enhance the activity on the other nuclease site (Figure 4). Furthermore, interactions with XPB block the N-terminal nuclease activity and change the properties of DNA incision by the Bax1 nuclease domain as the AfXPB–Bax1 complex shows different DNA incision patterns from DNA incision by AfBax1 alone (Figure 4B). Similarly, the TaXPB–Bax1 complex was previously reported to cleave 5′-overhang while TaBax1 alone cleaves 3′-overhang (40). These results together demonstrate protein-protein interactions regulate DNA incision by the Bax1 nuclease in order to remove a fragment of damage DNA during DNA repair (Figure 8).

Bax1 likely contains two active sites: one at the N-terminal domain for 5′ cleavage at the DNA bubble and the other at the nuclease domain for 3′ cleavage to the bubble (Figure 8). Formation of the XPB–Bax1 complex enhances the nuclease activity on the 3′ cleavage (like XPG in eukaryotes) by Bax1. When a bubble is created around DNA lesion during NER, the XPB–Bax1 complex is responsible for the 3′ incision to the damage while the 5′ incision to the damage is likely achieved by two different mechanisms. In euryarchaea lack of XPF (*like T. acidophilum)*, Bax1 is primarily a monomer and acts like XPF to cleave the damage strand 5′ to the lesion. In crenarchaea containing XPF homolog, the 5′-incision is likely carried out by XPF nuclease. In this case, Bax1 forms a homodimer to mask both active sites in order to avoid an active Bax1 nuclease for competition with XPF. This can be achieved by the two Bax1 monomers to interact with each other through N-terminal domain to C-terminal domain cross-interactions, resulting in both active sites at the nuclease domain and the N-terminal domain blocked from access by DNA substrates in the Bax1 homodimer (Figure 8). In agreement with this model, we observed that StBax1 forms a homodimer in solution (Figure 1C) and, in the crystal, the C-terminal StBax1 from a nearby heterodimer contacts the N-terminal StBax1 associated with StXPB (Supplementary Figure S4).

To our surprise, the StXPB–Bax1 complex fits very well with the Cryo-EM structure of the XPA-TFIIH core-forked DNA complex recently determined by Cramer and colleagues (34) although Bax1 has no sequence homology with either XPG or XPF/ERCC1 endonuclease. When the HD2 domain of StXPB is superimposed with the HD2 of human XPB in the Cryo-EM structure (Supplementary Figure S6), the rest of StXPB has no main chain clashes with the Cryo-EM structure. Furthermore, the StBax1 is positioned with the forked DNA to allow the nuclease domain of Bax1 to incise DNA at the ds-ss junction, suggesting XPG or XPF could interact similarly with XPB and the bubble DNA during eukaryotic NER.

## DATA AVAILABILITY

Atomic coordinates and structural factors for the structures for the reported crystal structures have been deposited with the Protein Data Bank under accession number 6P66 (AfXPB–Bax1 heterodimer) and 6P4O (StXPB–Bax1 heterodimer).

## Supplementary Material

gkaa324_Supplemental_Files
