# Supplementary material for: Structural basis of the XPB–Bax1 complex as a dynamic helicase–nuclease machinery for DNA repair
Source: Nucleic Acids Res. 2020 May 6;48(11):6326–39. doi: 10.1093/nar/gkaa324 (PMC7293015; doi:10.1093/nar/gkaa324)
Supplement: gkaa324_Supplemental_Files [file gkaa324_supplemental_files.zip › Nar2019supple_Fan_rev.pdf]

Title: Structural basis of the XPB-Bax1 complex as a dynamic helicase-nuclease machinery for DNA repair

Kevin DuPrez[1], Feng He[1], Zhenhang Chen, Eduardo Hilario, and Li Fan*

Department of Biochemistry, University of California, Riverside, CA 92521, USA.

[1]These authors contributed equally
*To whom correspondence should be addressed. Tel: 1-951-827-3630; Fax: 1-951-8274434;
Email: lifan@ucr.edu
ORCIDs: 0000-0002-6763-0467

Supplementary Data

Table S1. Statistics of crystallographic data and structural refinement for the XPB-Bax1 complex

**Dataset**	AfXPB:Bax1 Native	AfXPB:Bax1(SeMet) Peak	AfXPB:Bax1(SeMet) Inflection	AfXPB:Bax1(SeMet) Remote	StXPB:Bax1 Native
**Data collection**					
Space group	C 1 2 1	C 1 2 1	C 1 2 1	C 1 2 1	C 1 2 1
Cell dimensions:					
a, b, c (Å)	200.60, 129.84, 108.15	194.59, 129.78, 110.30	194.80, 129.94, 110.50	194.84, 130.11, 110.70	217.72, 125.85, 148.97
α, β, γ (°)	90.00, 104.30, 90.00	90.00, 103.92, 90.00	90.00, 103.92, 90.00	90.00, 104.03, 90.00	90.00, 101.52, 90.00
Resolution (Å)	40.00 – 3.00 (3.16 – 3.00)	29.04 – 3.15 (3.27 – 3.15)	29.09 – 3.15 (3.27 – 3.15)	29.14 – 3.15 (3.27 – 3.15)	30.00 – 3.15 (3.32 – 3.15)
R_{pim}	0.064 (0.623)	0.054 (0.685)	0.056 (0.859)	0.060 (0.912)	0.072 (0.397)
$I/\sigma I$	8.4 (1.4)	12.2 (1.7)	11.7 (1.4)	11.2 (1.3)	6.6 (1.7)
Completeness (%)	98.5 (98.4)	95.8 (97.4)	96.1 (97.4)	95.8 (97.3)	85.0 (79.5)
Multiplicity	3.5 (3.6)	6.3 (6.3)	6.3 (6.4)	6.3 (6.3)	4.5 (3.7)
CC1/2 (%)	99.7 (56.7)	99.8 (58.8)	99.9 (50.4)	99.9 (41.0)	99.1 (45.1)
**Refinement**					
Refinement program	PHENIX 1.15.2_3472				Refmac 5.8.0238
Resolution (Å)	39.93 – 3.00				29.93 – 3.15
No. reflections	52873				55900
R_{work} / R_{free}	22.5 (35.0) / 24.0 (38.2)				26.51 (40.7) / 28.24 (44.2)
Number of atoms	13249				20038
Protein	13136				19961
Ligands	3				21
Water	110				56
Ramachandran favored	94.31%				95.73%
Ramachandran allowed	5.69%				4.20%
Ramachandran outliers	0.00%				0.07%
R.m.s.d Bond length	0.006 Å				0.007 Å
R.m.s.d Bond angles	1.20°				1.33°
Fo, Fc correlation	0.94				0.90
Anisotropy	0.225				0.055
Averaged B factor	116.0 Å^2				78.86 Å^2

Values in parentheses are for highest-resolution shell.

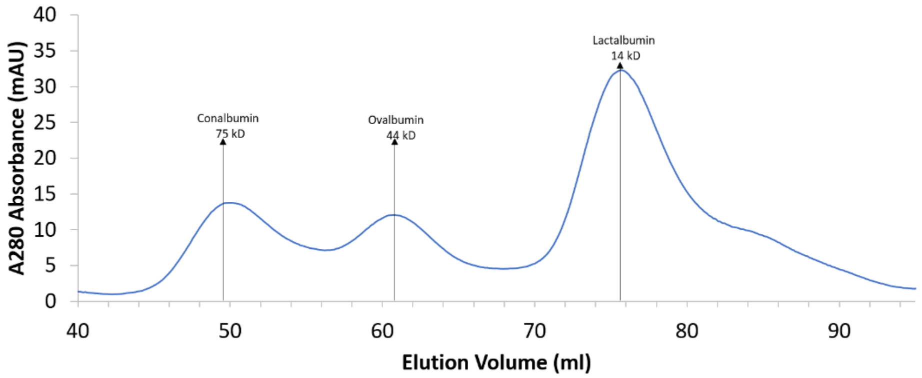

Figure S1. **S200 gel filtration chromatographic profile of three protein markers.** A mixture of protein markers Conalbumin (75 kDa), Ovalbumin (44 kDa), and Lactalbumin (14 kDa) from GE Healthcare was applied to the HiPrep 16/60 Sephacryl S-200 gel filtration column (GE Healthcare) following the manufacture's procedure for calibration.

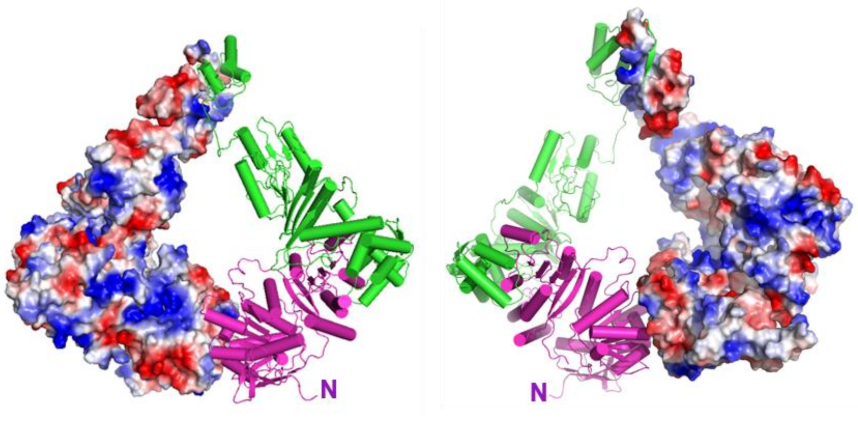

Figure S2. The electrostatic potential surfaces of the AfXPB-Bax1 heterodimer. One of the AfXPB-Bax1 heterodimers is presented as ribbons for reference. Electrostatic potential surfaces are calculated in Pymol (1) and are presented in relative scale. Negative surfaces are shown in red, positive surfaces are shown in blue.

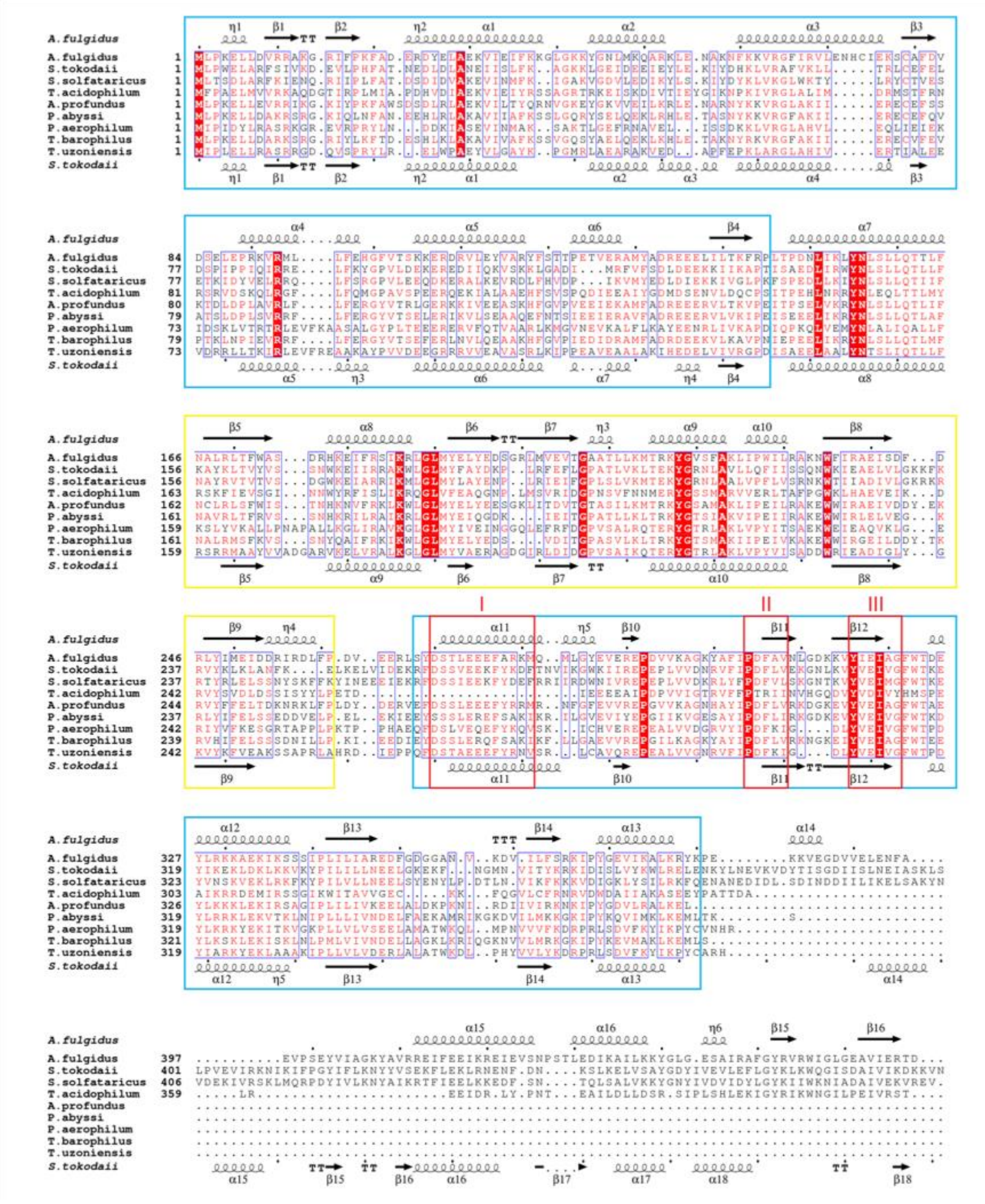

Figure S3. Sequence and structural alignments of Bax1 orthologues. Amino acid sequences of Bax1 orthologues from *Archaeoglobus fulgidus*, *Sulfurisphaera tokodaii*, *Saccharolobus solfataricus*, *Thermoplasma acidophilum*, *Archaeoglobus profundus*, *Pyrococcus abyssi*, *Pyrobaculum aerophilum*, *Thermococcus barophilus*, and *Thermoproteus uzoniensis* are aligned with a 0.5 threshold for similarity. Alignment was performed with Clustal Omega (2) and depicted using ESPript 3.0 server (3). Secondary structure elements for *A. fulgidus* (top) and *S. tokodaii* (bottom) are numbered and represented according to the PDB files for the AfXPB-Bax1 (PDB entry 6P66) and StXPB-Bax1 (PDB entry 6P4O) structures. Domains of Bax1 are colored in frames: NTD – cyan, CRD – yellow, NUS/nuclease motifs – cyan/red, except the C-terminal domain that is not conserved in all Bax1 orthologues. *S. tokodaii, S. solfataricus*, *P. aerophilum, T. uzoniensis* belong to the phylum Crenarchaeota while others belong to the phylum Euryarchaeota.

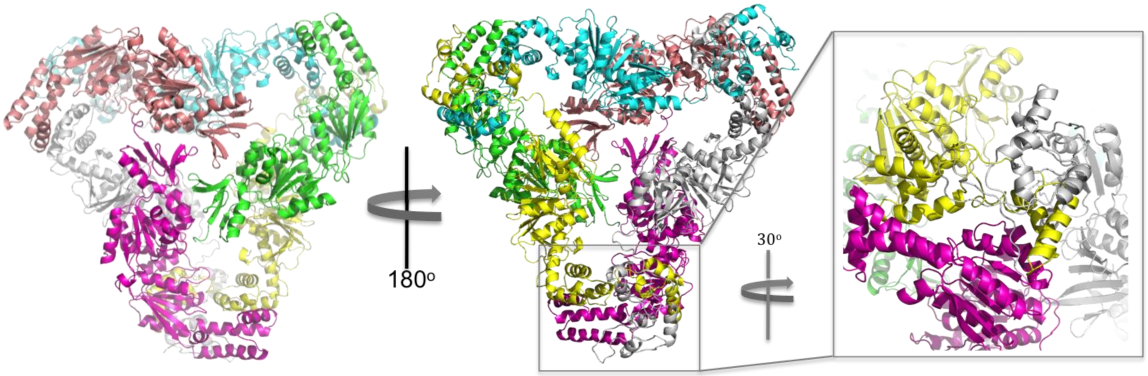

Figure S4. Three StXPB-Bax1 heterodimers in the asymmetric unit. StXPB molecules are presented in magenta, green, and wheat cartoons while StBax1 molecules are in gray, yellow, and cyan cartoons. Three StXPB-Bax1 heterodimers form a triangular shape in the asymmetric unit. At each angle (see Zoom-in insert for details), the C-terminal domain of adjacent Bax1 (gray) interacts with the N-terminal domain of Bax1 (yellow) complexed with XPB (magenta).

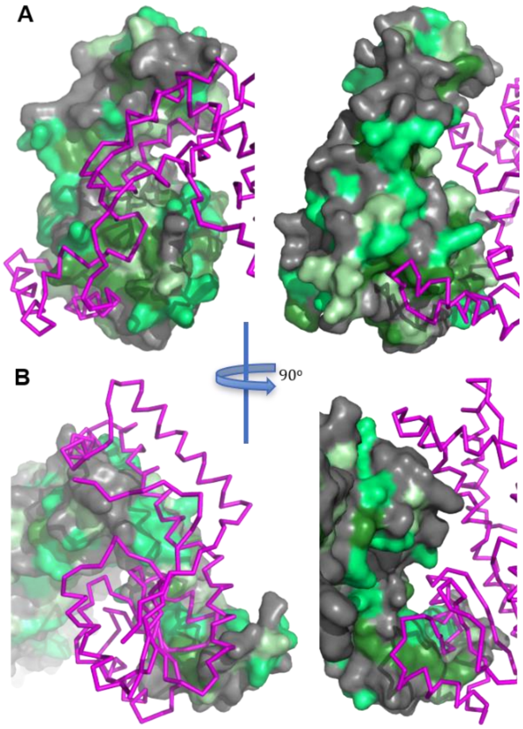

Figure S5. The XPB:Bax1 interaction interfaces are conserved among archaea. A. Sequence conservation on the surface of XPB interacting with Bax1(magenta ribbons). **B** Sequence conservation on the surface of Bax1 interacting with XPB (magenta ribbons). Surfaces are colored based on Clustal Omega (2) alignment of XPB sequences (A) or Bax1 sequences (B) from *S. tokodaii*, *A. fulgidus*, *S. solfataricus*, and *T. acidophilum*. Surfaces are color coded as identical residues in dark green, highly similar residues in green, similar residues in pale green, not conserved residues in gray.

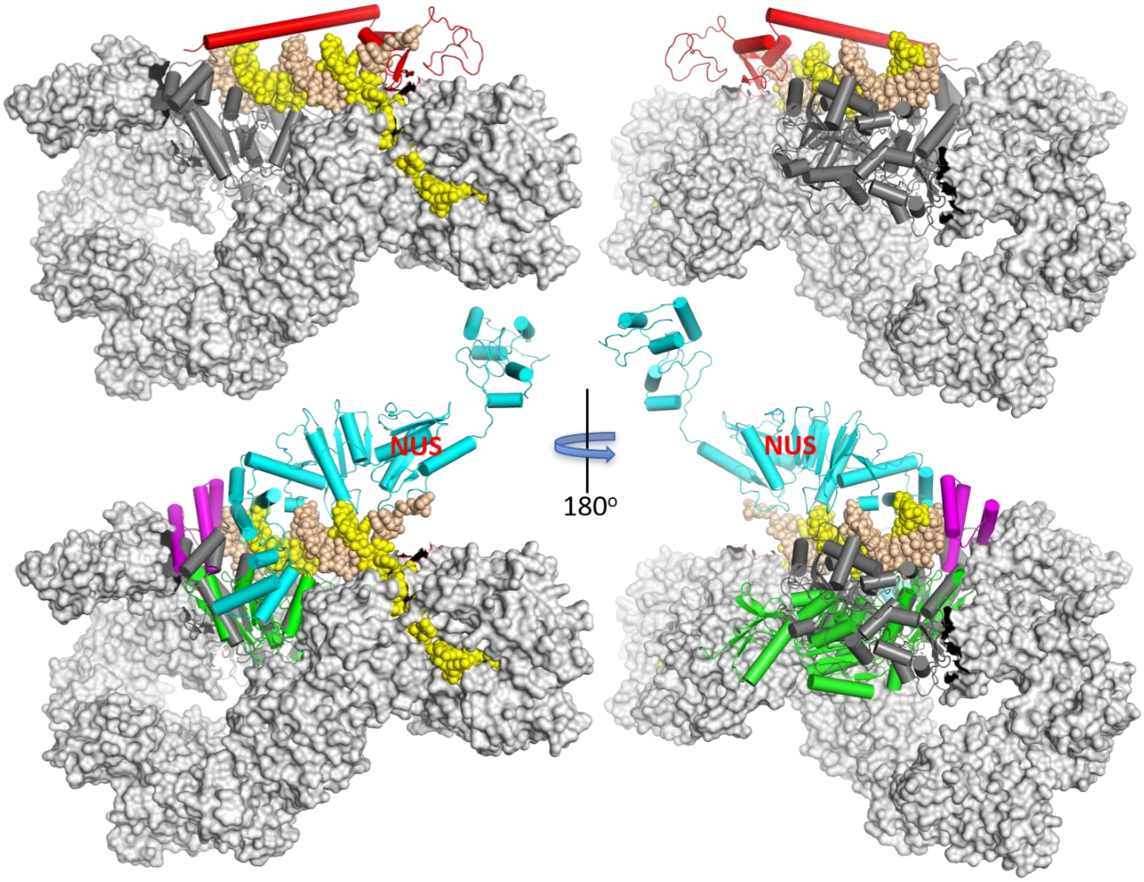

Figure S6. The StXPB-Bax1 complex fits very well with the Cryo-EM structure of the XPA-TFIIH core-forked DNA complex. Top: The Cryo-EM structure of human XPA-TFIIH core -forked DNA complex (PDB ID: 6RO4)(4). XPB is shown in grey cartons and the rest of TFIIH core is in light grey surfaces. XPA is presented by red cartons. The forked DNA is shown as spheres with the 3'-overhang strand in yellow and the 5'-overhang strand in wheat. Bottom: The crystal structure of the StXPB-Bax1 complex is docked to the Cryo-EM structure by superimposing the HD2 (residues 307-439) of StXPB over the HD2 (residues 536-678) of human XPB. The RMSD is 2.1 Å over 142 residues. StXPB is shown in green cartons with the THM motif highlighted in magenta. StBax1 is displayed as cyan cartons with the nuclease domain labeled by NUS. For simplicity, XPA is omitted.

REFERENCES

1. DeLano, W. (2002) The PyMOL Molecular Graphics System. .
2. Sievers, F., Wilm, A., Dineen, D., Gibson, T.J., Karplus, K., Li, W., Lopez, R., McWilliam, H., Remmert, M., Soding, J. *et al.* (2011) Fast, scalable generation of high-quality protein multiple sequence alignments using Clustal Omega. *Mol Syst Biol*, **7**, 539.
3. Robert, X. and Gouet, P. (2014) Deciphering key features in protein structures with the new ENDscript server. *Nucleic Acids Res*, **42**, W320-324.
4. Kokic, G., Chernev, A., Tegunov, D., Dienemann, C., Urlaub, H. and Cramer, P. (2019) Structural basis of TFIIH activation for nucleotide excision repair. *Nature communications*, **10**.
